# Mobility for maternal health among women in hard-to-reach fishing communities on Lake Victoria, Uganda; a community-based cross-sectional survey

**DOI:** 10.1186/s12913-021-06973-5

**Published:** 2021-09-10

**Authors:** Ali Ssetaala, Julius Ssempiira, Gertrude Nanyonjo, Brenda Okech, Kundai Chinyenze, Bernard Bagaya, Matt A Price, Noah Kiwanuka, Olivier Degomme

**Affiliations:** 1 UVRI-IAVI HIV Vaccine Program, Entebbe, Uganda; 2 Ghent University International Centre for Reproductive Health, Gent, Belgium; 3 School of Public Health, Makerere University College of Health Sciences, Kampala, Uganda; 4 IAVI, New York, USA; 5 Department of Epidemiology and Biostatistics, University of California at San Francisco, California San Francisco, USA

**Keywords:** Mobility, Distance, Childbirth, Women, Island, Fishing, Communities, Uganda

## Abstract

**Background:**

Maternal mortality is still a challenge in Uganda, at 336 deaths per 100,000 live births, especially in rural hard to reach communities. Distance to a health facility influences maternal deaths. We explored women’s mobility for maternal health, distances travelled for antenatal care (ANC) and childbirth among hard-to-reach Lake Victoria islands fishing communities (FCs) of Kalangala district, Uganda.

**Methods:**

A cross sectional survey among 450 consenting women aged 15–49 years, with a prior childbirth was conducted in 6 islands FCs, during January-May 2018. Data was collected on socio-demographics, ANC, birth attendance, and distances travelled from residence to ANC or childbirth during the most recent childbirth. Regression modeling was used to determine factors associated with over 5 km travel distance and mobility for childbirth.

**Results:**

The majority of women were residing in communities with a government (public) health facility [84.2 %, (379/450)]. Most ANC was at facilities within 5 km distance [72 %, (157/218)], while most women had travelled outside their communities for childbirth [58.9 %, (265/450)]. The longest distance travelled was 257.5 km for ANC and 426 km for childbirth attendance.

Travel of over 5 km for childbirth was associated with adolescent girls and young women (AGYW) [AOR = 1.9, 95 % CI (1.1–3.6)], up to five years residency duration [AOR = 1.8, 95 % CI (1.0-3.3)], and absence of a public health facility in the community [AOR = 6.1, 95 % CI (1.4–27.1)].

Women who had stayed in the communities for up to 5 years [AOR = 3.0, 95 % CI (1.3–6.7)], those whose partners had completed at least eight years of formal education [AOR = 2.2, 95 % CI (1.0-4.7)], and those with up to one lifetime birth [AOR = 6.0, 95 % CI (2.0-18.1)] were likely to have moved to away from their communities for childbirth.

**Conclusions:**

Despite most women who attended ANC doing so within their communities, we observed that majority chose to give birth outside their communities. Longer travel distances were more likely among AGYW, among shorter term community residents and where public health facilities were absent.

**Trial registration:**

PACTR201903906459874 (Retrospectively registered). https://pactr.samrc.ac.za/TrialDisplay.aspx?TrialID=5977.

**Supplementary Information:**

The online version contains supplementary material available at 10.1186/s12913-021-06973-5.

## Background

Uganda still has a high number of women dying from pregnancy and childbirth related complications, with a maternal mortality ratio of 336 maternal deaths per 100,000 live births [1]. Most of these deaths are preventable, implying that the country is still in need of innovative cost-effective ways of reducing maternal deaths [2, 3].

Skilled attendance during every pregnancy and childbirth is one of the potential ways of reducing maternal morbidity and deaths [4, 5]. Skilled birth attendance is affected by maternal health services physical, financial accessibility and socio-cultural acceptability [6]. Physical access to maternal health services influences women’s skilled birth attendance. Availability of good quality health services within realistic reach of women who need them, including working hours that permit women to get services whenever needed, is key to skilled birth attendance [7]. Women’s ability to cater for transport expenses without fiscal difficulty, including cost of taking time off work and cost of health services, affects financial access to maternal health care. Skilled birth attendance may also be affected by acceptability of maternal health services provided or women’s willingness to seek services. Women may perceive the maternal health services to be ineffective and socio-culturally deterring. Issues like provider’s age, gender, religious and tribal affiliation may deter women’s access to skilled birth attendance. Access to skilled birth attendance is still a challenge despite improvements over the years, especially in rural hard to reach Ugandan communities [8, 9].

Distance from one’s household to health care affects physical access to health facility for ANC and skilled birth attendance, as most deaths occur in resource limited hard to reach rural settings that are far away from quality maternal health care and other social services [7, 10–14]. Distance from residence to the health facility has been linked to facility births, maternal, neonatal and child health mortality [7, 13, 15–22]. In some settings, living further away from the health facility was associated with increased early neonatal deaths, while proximity to a health facility was linked to better neonatal survival [15, 23]. Staying closer to health facility was associated with fewer still births among women in some settings [16]. Improving quality of maternal health care at health facilities closer to where women live has shown to reduce mortality and allow continuity of care [24].

Availability of services at the nearby facilities influences travel distance from women’s households to the health facilities for skilled maternal health care. If services are unavailable at nearby facilities, women are more likely to travel to far off facilities that have the services or attend unskilled maternal health care. Communities and women make their own assessments of quality maternal health care at a given facility, which also influences mobility for maternal health [13]. Majority of the population in Uganda lives within 5 km to a health facility, with varying proportions based whether one is living in an urban or rural area [8]. However physical accessibility may not directly translate into receipt of quality maternal health services as some of these health facilities lack skilled staff, staff accommodation, emergency obstetric care medicines, equipment, and surgical facilities, which exacerbates women’s travel for maternal health [25–28]. Travel for maternal health may also be precipitated by domestic violence, household poverty, women’s lack of knowledge on maternal health-related issues, quality, and availability of services at a given health facility [28, 29]. Having childbirths from health facilities not closest to the mother’s home (mobility for maternal health or bypassing health facilities) has been previously noted in some settings, ranging between 42 and 75 % [25, 26, 28], though unknown for FCs on Lake Victoria, Uganda.

Women in FCs on Lake Victoria are highly mobile, often travelling away from access to healthcare [30]. Fishing communities being rural hard to reach resource limited settings, women’s mobility might affect skilled birth attendance, maternal morbidity, and mortality. Women mainly travel for work related reasons, some travel for maternal health often in circular patterns between the FCs and the mainland [30].

The level of mobility, distances travelled for maternal health and associated factors among women in FCs is not known. We therefore explored women’s mobility for maternal health, distances to childbirth attendance and associated factors to inform targeted policies for childbirth interventions to improve maternal health among these hard-to-reach islands FCs in Kalangala district, Uganda.

## Methods

### Study design, setting and population

During January to May 2018, we enrolled women into a cross-sectional survey, selected based on age (15 to 49 years at survey time), being pregnant or history of a pregnancy outcome (live birth, still birth or abortion) in the past 6 months. The survey was part of an intervention aimed at improving maternal health through capacity strengthening of community health workers. Women were enrolled from six purposively selected hard to reach Ugandan islands in Kalangala district. The islands were selected from 12 islands where the authors had previous research experience based on being most hard-to-reach [31]. Exhaustive methods are published elsewhere [31, 32].

In this study we focused on those women who reported a previous birth, to understand their mobility for ANC, childbirth, distances travelled and associated factors.

We collected global positioning system (GPS) coordinates (latitude and longitude) for women’s baseline household locations using open data kit (ODK) collect [33]. Women were asked the names of health facilities where they accessed ANC and childbirth services for the most recent birth. If a woman attended more than one health facility, the highest-level facility was considered. Health facilities GPS coordinates were documented using Google maps. Mapping of households and health facilities was done using Quantum Graphic Information System (QGIS) software version 3.16.3 with a coordinate reference system (CRS) of world geodetic system (WGS) 84, geodetic parameter dataset (EPSG) code number 4326 [34]. Straight-line distances in kilometers (km) between each woman’s household location and the facility they attended for ANC and or childbirth were calculated by QGIS distance matrix, using the Universal Transverse Mercator (UTM) CRS of standardized WGS 84, EPSG code number 32,636 [34, 35]. Straight-line distance despite being a less accurate (does not account for environmental conditions, time and effort that might impact on the real distance) measure of distance travelled, this method provides a suitable alternative and has been previously used to assess ease of access to health services in remote settings [36–39]. Household to ANC facility distances were complete for 218 women, while 250 women had household to childbirth location distances completed, and these were used in the distance to ANC and birth attendance analysis.

### Statistical methods

This analysis aimed at answering the following questions:

1. How far do women in fishing communities travel for ANC and childbirth services?
2. What factors are associated with a travel distance of over 5 km during the most recent childbirth?
3. What factors are associated with mobility for the most recent childbirth?

#### Study variables

The primary dependent variable was mobility for birth attendance, dichotomized into whether or not a woman had the most recent birth within or outside her community of residence.

Distance from household to birth facility was also a dependent variable, dichotomized into whether a woman moved up to 5 km or over 5 km from her household to a childbirth facility during the most recent childbirth.

Women’s socio-demographic characteristics were summarized using frequency tables and compared with the dependent variables (mobility for birth attendance and distance from household to ANC or childbirth facility within 5 km and over 5 km), using chi-square and Fisher Exact tests for categorical variables and median, range for continuous variables. We defined adequate distance to maternal health facility as having travelled within 5 km from the women’s households [40]. The selection of 5 km is based on previous work in low- and middle-income countries, indicating that being within 5 km of obstetric care facilities was related to heath facility births [41–43]. The Uganda Health Sector Development plan 2015/16 to 2019/20 also aimed at improving access to health through ensuring that at least 85 % of the population are within 5 km access to a health facility [40]. Uganda’s current strategy for improving health service delivery also involves upgrading and construction of health facilities at subcounty level to attain a within 5 km walking distance to a health facility [44].

Adjusted odds ratios (AOR) of mobility for birth attendance and distance to childbirth facility were estimated using multivariable logistic regression modeling, testing for associations with the independent variables. A priori selection of independent variables to include in the multivariable models was based on previous literature and biological plausibility. Independent variables included in the bivariable analysis were residence community with or without a public health facility, age groups, duration of community stay, religious affiliation, marital status, highest education, partner’s highest education, main occupation, participant’s health decisions maker, lifetime births, pregnancy planned, history of pregnancy loss, number of ANC visits, at least four ANC visits attendance, receipt of ANC components, skilled birth attendance, and type of childbirth facility. Additionally, those variables found to have a bivariable statistical significance at an alpha (α) of ≤ 0.2 were included. The final best suited independent variables in the model were those with the lowest P-value, lowest model Akaike’s information criterion and Bayesian information criterion values. All analyses were done using STATA® version 15 [45]. Tables were created using asdoc, a STATA program written by Shah [46]. Strengthening the reporting of observational studies in epidemiology (STROBE) guidelines for cross sectional studies were followed in this article [47].

## Results

### Participants’ characteristics

The survey involved 486 women of whom 450 (92.6 %) had a previous childbirth. Among those with a previous childbirth, age ranged between 15 and 45 (median, 27) years. Over a third of participants were AGYW aged 15–24 years [36.2 %, (163/450)]. Majority of the women had never studied beyond seven years of formal education [70.4 %, (317/450)], had stayed in the communities for up to five years [73.8 %, (332/450)], residing in communities with a government (public) health facility [84.2 %, (379/450)]. See Table 1.

**Table 1:** Participants characteristics by mobility for birth attendance among 450 women residing in 6 island fishing communities in Uganda

Characteristic	Total (%)	Mobility for skilled birth (%)		*p*-value
		No mobility	Yes, moved	
All women	450	185 (41.1)	265 (58.9)	
Age; range (median) years	15–45 (27)	15–45 (29)	16–43 (26)	
**Age groups**				< 0.05
25–49	287 (63.8)	137 (74.0)	150 (56.6)	
15–24	163 (36.2)	48 (26.0)	115 (43.4)	
**Duration of community stay**				< 0.05
> 5 years	118 (26.2)	63 (34.0)	55 (20.7)	
≤ 5 years	332 (73.8)	122 (66.0)	210 (79.3)	
**Religion**				0.35
Muslim	95 (21.1)	42 (22.7)	53 (20.0)	
Catholic	191 (42.4)	71 (38.4)	120 (45.3)	
Others	164 (36.4)	72 (38.9)	92 (34.7)	
**Highest education (Years)**				0.03
≥ 8	133 (29.6)	44 (23.8)	89 (33.6)	
1–7	285 (63.3)	123 (66.5)	162 (61.1)	
0	32 (7.1)	18 (9.7)	14 (5.3)	
**Lifetime births**				< 0.05
Over one	331 (73.6)	159 (85.9)	172 (64.9)	
Up to one	119 (26.4)	26 (14.1)	93 (35.1)	
**Community public health facility**				0.21
Present	379 (84.2)	151 (81.6)	228 (86.0)	
Absent	71 (15.8)	34 (18.4)	37 (14.0)	
**Pregnancy planned**				0.06
No	72 (34.3)	30 (42.9)	42 (30.0)	
Yes	138 (65.7)	40 (57.1)	98 (70.0)	
**Ever lost pregnancy**				0.94
No	252 (56.0)	104 (56.2)	148 (55.8)	
Yes	198 (44.0)	81 (43.8)	117 (44.2)	
**ANCx4 attendance**				0.04
Yes	137 (30.4)	66 (35.7)	71 (26.8)	
No	313 (69.6)	119 (64.3)	194 (73.2)	
**All seven ANC components receipt**				0.90
No	393 (87.3)	162 (87.6)	231 (87.2)	
Yes	57 (12.7)	23 (12.4)	34 (12.8)	
**Most recent birth attendance**				< 0.05
Unskilled	59 (13.1)	51 (27.6)	8 (3.0)	
Skilled	391 (86.9)	134 (72.4)	257 (97.0)	
**Birth facility**				< 0.05
Private	126 (28.0)	36 (19.4)	90 (34.0)	
Government Hospital	109 (24.2)	0 (0.0)	109 (41.1)	
Government Health Centre	154 (34.2)	93 (50.3)	61 (23.0)	
Home	51 (11.3)	46 (24.9)	5 (1.9)	
TBA	10 (2.2)	10 (5.4)	0 (0.0)	

Most women never attended at least four ANC visits during the recent childbirth [69.6 %, (313/450)]. Most of the childbirths were attended to by a skilled health worker [86.9 %, (391/450)], mainly occurring at government health facilities [58.4 %, (263/450)], away from maternal health facilities nearest to participants’ residences [86 %, (228/265)]. See Table 1.

### Mobility for Maternal health

#### Distance to ANC facility

**Fig. 1:**
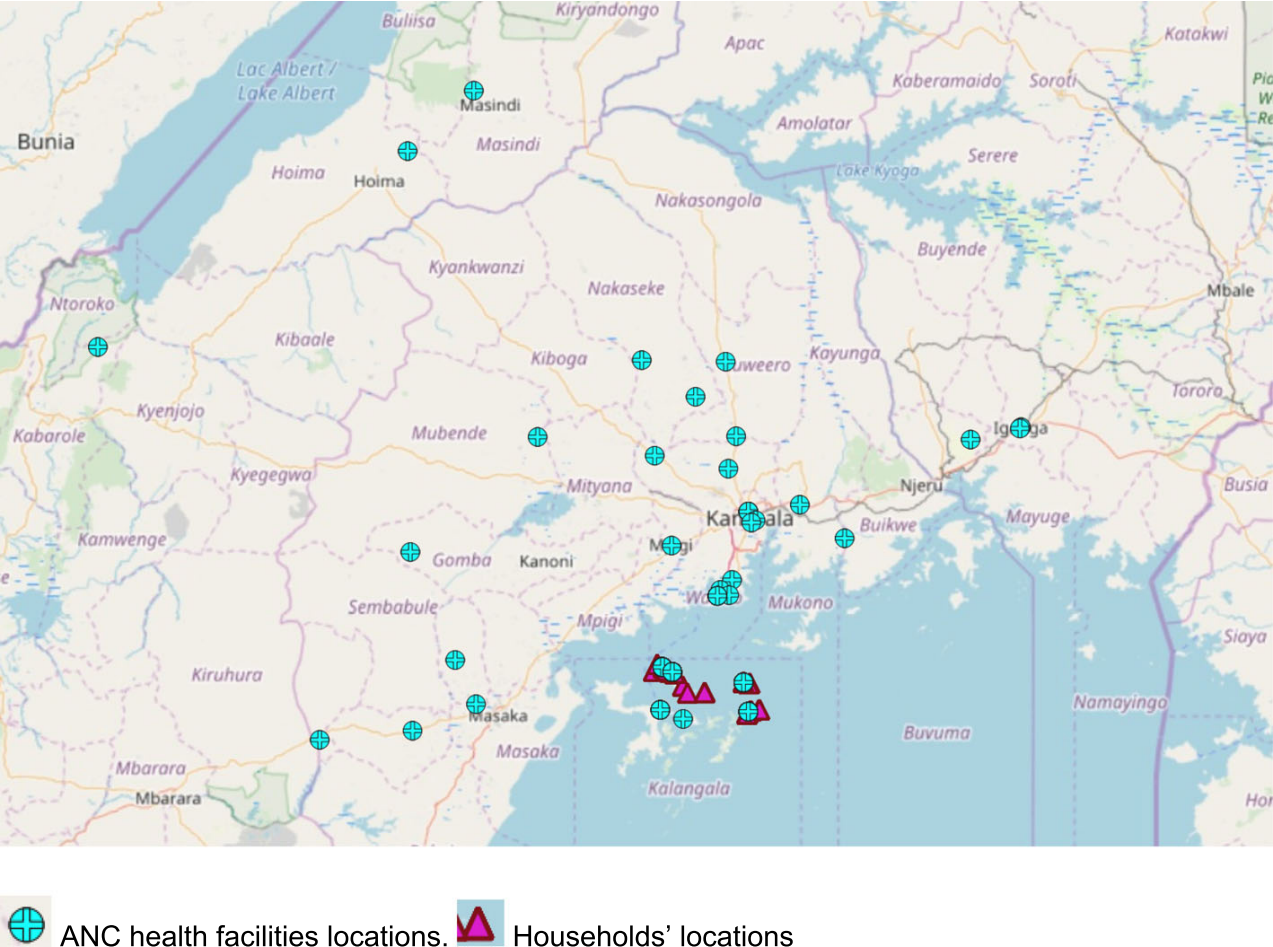
Locations of health facilities where the women received ANC.

The distance from a woman’s home to the ANC facility of choice ranged from less than 1 to 257.5 km, with a median of 2.2 km. Women who attended ANC travelled much shorter distances than those who had a childbirth (median distance 2.2 km vs. 44.9 km). The majority of women attended ANC at facilities within 5 km from their households [72 %, (157/218)], however nearly a third of participants attended ANC at facilities over 5 km from their households [28 %, (61/218)]. Over three quarters of women who were living in communities with a public health facility attended ANC facilities within 5 km from their households [79.3 %, (157/198)]. See Fig. 1; Table 2.

**Table 2:** Women’s characteristics by distance to ANC and birth attendance

Characteristic	Total (%)	ANC distance (%)		*p*-value	Total (%)	Birth distance (%)		*p*-value
		≤ 5 km	> 5 km			≤ 5 km	> 5 km	
All women	218	157 (72.0)	61 (28.0)		250	86 (34.4)	164 (65.6)	
Range (Median)		0-257.5 (2.2) km				0-426 (44.9) km		
Age; range (median) years	16–45 (27)	16–45 (27)	16–41 (26)		15–45 (27)	15–45 (29)	17–43 (26)	
**Age groups**				0.61				< 0.05
25–49	138 (63.3)	101 (64.3)	37(60.7)		156 (62.4)	64 (74.4)	92 (56.1)	
15–24	80 (36.7)	56 (35.7)	24 (39.3)		94 (37.6)	22 (25.6)	72 (43.9)	
**Duration of community stay**				0.71				< 0.05
> 5 years	64 (29.4)	45 (28.7)	19 (31.2)		79 (31.6)	42 (25.6)	37 (43.0)	
≤ 5 years	154 (70.6)	112 (71.3)	41 (68.8)		171 (68.4)	122 (74.4)	49 (57.0)	
**Highest education (Years)**				0.26				0.37
≥ 8	65 (29.8)	42 (26.7)	23 (37.7)		71 (28.4)	22 (25.6)	49 (29.9)	
1–7	136 (62.4)	103 (65.6)	33 (54.1)		158 (63.2)	54 (62.8)	104 (63.4)	
0	17 (7.8)	12 (7.6)	5 (8.2)		21 (8.4)	10 (11.6)	11 (6.7)	
**Occupation**				1.00				0.37
Housewife	112 (51.4)	82 (52.2)	30 (49.2)		123 (49.2)	37(43.0)	86 (52.4)	
Fishing related	13 (6.0)	6 (3.8)	7 (11.5)		13 (5.2)	5 (5.8)	8 (4.9)	
Others	93 (42.6)	69 (44.0)	24 (39.3)		114 (45.6)	44 (51.2)	70 (42.7)	
**Health decisions maker**				0.08				0.49
Others	117 (53.7)	90 (57.3)	27 (44.3)		129 (51.6)	47 (54.6)	82 (50.0)	
Respondent and Partner	101 (46.3)	67 (42.7)	34 (55.7)		121 (48.4)	39 (45.4)	82 (50.0)	
**Community public health facility**				< 0.05				< 0.05
Present	198 (90.8)	157 (100)	41 (67.2)		226 (90.4)	84 (97.7)	142 (86.6)	
Absent	20 (9.2)	0 (0.0)	20 (32.8)		24 (9.6)	2 (2.3)	22 (13.4)	
**ANCx4 attendance**				0.32				< 0.05
Yes	101 (46.3)	76 (48.4)	25 (41.0)		94 (37.6)	43 (50.0)	51 (31.1)	
No	117 (53.7)	81 (51.6)	36 (59.0)		156 (62.4)	43 (50.0)	113 (68.9)	
**Lifetime births**				1.00				< 0.05
Over one	164 (75.2)	118 (75.2)	46 (75.4)		191 (76.4)	78 (90.7)	113 (68.9)	
One	43 (19.7)	31 (19.7)	12 (19.7)		55 (22.0)	6 (7.0)	49 (29.9)	
None	11 (5.1)	8 (5.1)	7 (4.9)		4 (1.6)	2 (2.3)	2 (1.2)	

### Distance to childbirth attendance and associated factors

**Fig. 2:**
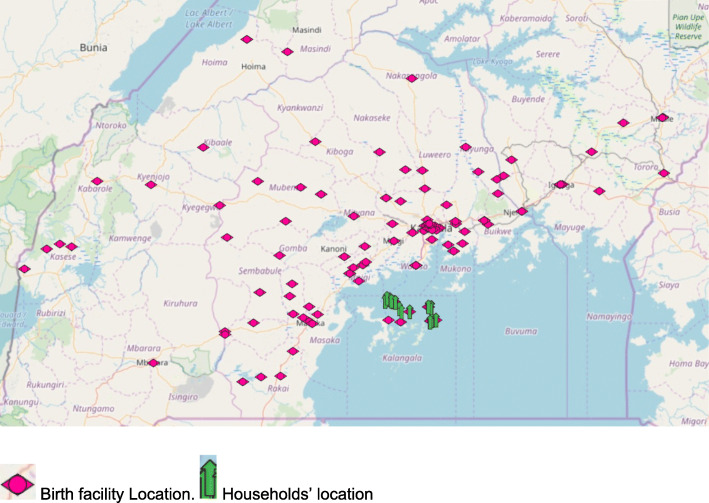
Locations of health facilities where the women had childbirths.

Distance to birth facility ranged from less than 1 to 426 km, with a median 44.9 km. More than six in ten women travelled over 5 km from their residences for childbirth [65.6 %, (164/250)]. See Table 1. Women who were residing in communities without a public health facility were 6 times as likely as those who were in communities with a public health facility to have had childbirth attendance more than 5 km from their households [AOR = 6.1, 95 % (1.4–27.1)]. AGYW were twice as likely as their older counterparts to have had a childbirth attendance more than 5 km from their residences [AOR = 1.9, 95 % CI (1.0-3.6)], as well as women who had stayed in the communities for up to 5 years [AOR = 1.8, 95 % CI (1.0-3.3)]. Women were also less likely to have had a childbirth attendance at facilities more than 5 km from their households with each additional ANC visit attended [AOR = 0.9, 95 % CI (0.8-1.0)]. See Table 3; Fig. 2.

**Table 3:** Factors associated with over 5 km distance to birth attendance

Distance to birth attendance	COR	95 % CI	AOR	95 % CI
Community public health facility				
Present	(Ref)		(Ref)	
Absent	6.5	1.5–28.4	6.1	1.4–27.1
Education (Years)				
0	(Ref)			
1–7	1.8	0.7–4.4		
≥ 8	2.0	0.7–5.5		
Age groups (Years)				
25–49	(Ref)		(Ref)	
15–24	2.3	1.3-4.0	1.9	1.1–3.6
Duration of community stay				
> 5 years	(Ref)		(Ref)	
≤ 5 years	2.2	1.3–3.8	1.8	1.0-3.3
ANC times	0.9	0.8-1.0	0.9	0.8-1.0

### Mobility for birth attendance

The majority of women moved outside their communities to seek childbirth attendance away from their residential communities [58.9 %, (265/450)]. Women who moved for birth attendance were younger (range 16–43 years, median 26 years) and had stayed in the communities for up to five years [79.3 %, (210/265)]. Majority of the women who moved had not attended at least four ANC visits during the most recent birth [73.2 %, (194/265)], despite having planned for their most recent pregnancies [70 %, (98/140)]. See Table 1.

Over eight in ten women who had birth attendance outside their residential communities had by-passed public maternal health facilities within their communities [86 %, (228/265)], with over half of women residing in communities without a public health facility having travelled for birth attendance [52.1 %, (37/71)]. See Table 1.

### Factors associated with mobility for birth attendance

Women who were residing in communities with a public health facility were twice as likely to have moved for skilled birth attendance than those in communities without public health facilities [AOR = 2.1, 95 % CI (0.8–5.3)]. See Table 4.

**Table 4:** Factors associated with mobility for birth attendance

Characteristic	COR	95 % CI	AOR	95 % CI
Community public health facility				
Absent	(Ref)		(Ref)	
Present	1.4	0.8–2.3	2.1	0.8–5.3
Age groups (Years)				
15–24	(Ref)			
25–49	0.5	0.3–0.7	1.8	0.7–4.7
Duration of community stay				
> 5 years	(Ref)		(Ref)	
≤ 5 years	2.0	1.3-3.0	3.0	1.3–6.7
Occupation				
None-Housewife	(Ref)		(Ref)	
Housewife	1.4	0.9-2.0	1.5	0.8-3.0
Partner education (Years)				
0–7	(Ref)			
≥ 8	1.7	1.2–2.6	2.2	1.0-4.7
Pregnancy planned				
No	(Ref)			
Yes	1.8	1.0-3.2	1.2	0.6–2.5
ANC times	0.9	0.9-1.0	0.9	0.7–1.2
Lifetime births				
Over one	(Ref)		(Ref)	
Up to one	3.3	2.0-5.4	6.0	2.0-18.1

Women who had stayed in these island communities for up to five years were thrice as likely to have had birth attendance outside their residential communities than those who had stayed for over five years [AOR = 3.0, 95 % CI (1.3–6.7)]. Mobility for childbirth attendance decreased with each ANC visit attended at the most recent pregnancy [AOR = 0.9, 95 % CI (0.7–1.2)]. See Table 4.

Participants whose partners had completed at least eight years of formal education [AOR = 2.2, 95 % CI (1.0-4.7)], and those with up to one lifetime birth [AOR = 6.0, 95 % CI (2.0-18.1)] were more likely to have moved for childbirth attendance. See Table 4.

## Discussion

Majority of ANC attendance was at facilities within 5 km of the participants’ residences. Mobility for childbirth was common, many participants had by-passed public maternal health facilities within their communities, travelling over 5 km from their households. Longer distances (over 5 km) for childbirth were more likely among AGYW, shorter term community residents, and where public health facilities were absent.

Mobility for birth attendance might have been due to a lack of childbirth related trust in the available public health facilities within these communities. Our previous work indicated that components of ANC receipt by women in these FCs was low despite attending ANC, which might have prompted women to seek birth attendance from facilities outside their residential communities [32]. These findings are contrary to similar work in Tanzania which indicated that women living far from hospitals used primary health care facilities more [13].

Women who moved were slightly younger than those who did not move out of their residential communities for maternal health. Younger women are often new to the communities and inexperienced on maternal health related issues, not knowing where to seek care within these communities. This may encourage them to move to facilities outside their communities for maternal health services, especially to facilities near their parental homes. Young women are more prone to complications during pregnancy and childbirth and might have moved due to complications related to the pregnancy or childbirth. We however did not ascertain history of birth complications among study participants.

Women moved shorter distances for ANC attendance than for childbirth attendance. Women might have perceived facilities within their communities as being unable to provide skilled birth attendance after attending ANC from these facilities. Maternal health facilities within the participants’ communities might have been better equipped for ANC attendance than for skilled birth attendance, prompting women to move further for childbirth attendance. Absence of a public health facility in the community was associated with increased likelihood of a childbirth more than 5 km from the women’s households. This may imply that women in these communities still trusted public maternal health facilities for ANC and skilled birth attendance, despite probably not being fully equipped. These findings are consistent with previous work which indicated that women who lived farther away from a health facility had lower odds of facility births [10]. Targeted policies to increase presence of public health facilities in these islands FCs may help improve maternal health in these settings.

Having stayed in these FCs for up to five years was associated with increased likelihood of mobility for maternal health and having travelled over 5 km for childbirth attendance. Such women are relatively new to the communities with limited social support from friends and family. They might have opted for childbirths at locations closer to friends and family to ease support during childbirth. We did not collect information on whether the health facilities women moved to for childbirth were closer to their family or parental homes.

Women whose partners had attained eight or more years of formal education were likely to have travelled for childbirth attendance than those with less educated partners. Partners often provide support for childbirth assistance, including advice on where to seek care. Educated partners might have been more informed on pregnancy danger signs, location of quality obstetric care and could have advised their women to travel away from residential communities for better childbirth attendance. Educated partners are often socio-economically well off, capable of providing financial support for their women to travel for birth assistance. Education has been previously linked to skilled birth attendance and better maternal health [48–51].

Mothers with up to one lifetime childbirths were more likely to have travelled for maternal health. This is likely so because women with up to one lifetime childbirths might have perceived themselves to be at heightened risk for maternal health complications and opted to travel out of their communities for the most recent birth attendance. Similar findings were noted in other settings [26, 28].

Women who attended fewer ANC visits during the most recent birth were more likely to have travelled for maternal health, including moving over 5 km for childbirth attendance. The nationally recommended ANC visits is four [52]. Women who attended fewer ANC visits might have perceived themselves to be at high risk for childbirth complications and travelled seeking better services. Women who attended more ANC visits could have received more information about the importance and availability of skilled birth services at the health facilities within 5 km from their households and realized that there is no need to travel to far off facilities for childbirth. Health facilities in these islands FCs might have referred women who attended fewer ANC visits for fear of complicated childbirths, since they could have known little about their obstetric history. This adds to similar findings in other African settings [25, 53]. However similar work from Eastern Uganda indicated that attending ANC visits was associated with deliveries from far off health facilities, probably because nearby health facilities were not providing obstetric care [25]. Others did not find an association between ANC attendance and distance to a health facility [13, 54].

These study findings are generalizable to women with prior birth in these islands FCs. The results have implications for maternal health programs promoting skilled birth attendance among women with a previous birth in these islands hard to reach FCs. Efforts to encourage women attend ANC visits, availing public health facilities within these FCs might reduce distances travelled for skilled birth attendance.

Study limitations included the use of self-reports which may be affected by social desirability reporting. This was minimized through use of a study team that is well known, not from within and trusted in these FCs. Medical records were not accessed to compare with the self-reports as women had skilled births from different locations. We could not ascertain which women were referred to higher level health facilities due to pregnancy or birth complications. We did not ascertain the reasons for women’s travelling to facilities outside their residential communities. Access to maternal health care can also be affected by financial affordability and acceptability of the services, we did not assess these factors [55]. We did not collect data on the quality of roads, means of transport or effect of seasonal weather changes on the distance travelled by women. The findings are not generalizable beyond women with a prior childbirth in these islands FCs.

Despite the above limitations, this study provides crucial information on mobility for maternal health among women with a previous birth in these hard-to-reach islands FCs, which might inform programming and policies on provision of maternal health care in these settings.

## Conclusions

Despite most women who attended ANC doing so within their communities, we observed that mobility for childbirth was common, with majority having childbirths outside their communities. We found that longer distances for childbirth were likely among AGYW, shorter term FCs residents and where public health facilities were absent. Targeted policies to improve availability of public health facilities may reduce distances travelled for birth attendance in these hard-to-reach islands FCs’ settings.

## Supplementary Information

**Additional file 1:**

**Additional file 2:**

**Additional file 3:**

**Additional file 4:**

**Additional file 5:**

**Additional file 6:**

**Additional file 7:**

**Additional file 8:**

## Data Availability

All data generated or analyzed during this study are included in this article and its supplementary information files.

## Abbreviations

ANC: Antenatal Care
AOR: Adjusted Odds ratio
CI: Confidence Interval
COR: Crude Odds ratio
CRS: Coordinate Reference System
EPSG: Geodetic parameter dataset
FCs: Fishing Communities
GPS: Global positioning system
ICRH: International Center for Reproductive Health
ODK: Open Data Kit
STROBE: Strengthening the reporting of observational studies in epidemiology
UTM: Universal Transverse Mercator
UVRI: Uganda Virus Research Institute
WGS: World geodetic system
